# Poly(Vinyl Chloride) Spheres Coated with Graphene Oxide Sheets: From Synthesis to Optical Properties and Their Applications as Flame-Retardant Agents

**DOI:** 10.3390/polym13040565

**Published:** 2021-02-14

**Authors:** Mihaela Baibarac, Luiza Stingescu, Malvina Stroe, Catalin Negrila, Elena Matei, Liviu C. Cotet, Ion Anghel, Ioana E. Şofran, Lucian Baia

**Affiliations:** 1 National Institute of Materials Physics, Lab. Optical Processes in Nanostructured Materials, P.O. Box MG-7, R077125 Bucharest, Romania; luiza.stingescu@infim.ro (L.S.); malvina@infim.ro (M.S.); 2 National Institute of Materials Physics, Nanoscale Condensed Matter Laboratory, P.O. Box MG-7, R077125 Bucharest, Romania; catalin.negrila@infim.ro; 3 National Institute of Materials Physics, Multifunctional Materials and Structures Laboratory, P.O. Box MG-7, R077125 Bucharest, Romania; elena.matei@infim.ro; 4 Department of Chemical Engineering, Faculty of Chemistry and Chemical Engineering, “Babes-Bolyai” University, Arany Janos 11, 400028 Cluj-Napoca, Romania; cosmin.cotet@ubbcluj.ro; 5 “Alexandru Ioan Cuza” Police Academy, Fire Officers Faculty, Morarilor Str. 3, 022451 Bucharest, Romania; ion_anghel2003@yahoo.com (I.A.); ioana_sofran@yahoo.com (I.E.S.); 6 Faculty of Physics, “Babeș-Bolyai” University, Mihail Kogălniceanu 1, 400084 Cluj-Napoca, Romania; lucian.baia@phys.ubbcluj.ro

**Keywords:** poly(vinyl chloride), graphene oxide, vibrational properties, flame-retardant material

## Abstract

A new method to obtain poly(vinyl chloride) (PVC) spheres, which consists of an interaction between commercial PVC grains and hexyl ethyl cellulose and lauroyl peroxide at a temperature of 60 °C, is reported. The addition of the graphene oxide (GO) sheets dispersed in dimethylformamide to the reaction mixture leads to the generation of composites made of PVC spheres coated with GO sheets. Scanning electron microscopy studies have demonstrated that this method allows for the transformation of PVC grains with sizes between 75 and 227 μm into spheres with sizes varying from 0.7 to 3.5 μm when the GO concentration in the PVC/GO composite mass increases from 0.5 to 5 wt.%. Our studies of Raman scattering and FTIR spectroscopy highlight a series of changes that indicate the appearance of ClCH=CH–, CH_2_=CCl–, and/or –CH=CCl– units as a result of PVC partial dehydrogenation. New –COO– and C–OH bonds on the GO sheet surfaces are induced during the preparation of PVC spheres coated with GO sheets. A photoluminescence (PL) band with a maximum at 325 nm is reported to characterize the PVC spheres. A PVC PL quenching process is demonstrated to be induced by the increase in the concentration of the GO sheets in the PVC/GO composite mass. The perspectives regarding the use of this composite as a flame-retardant material are also reported.

## 1. Introduction

Over the last ten years, carbon-based nanostructures, such as graphene, graphene oxide (GO), and reduced graphene oxide, as well as their composites with macromolecular compounds, have received considerable attention [1]. GO gained the attention of scientific researchers due to its outstanding properties when compared to graphene and its potential as a promising precursor for the synthesis of the two-dimensional carbon derivative. It is defined as a monolayer of carbon atoms containing hexagonal rings with different functional groups (e.g., epoxy, hydroxyl, carbonyl, and carboxyl groups) covalently attached to the base plane and on the edges [2]. Due to the presence of these oxygen-containing groups, GO can be dispersed in polar solvents, such as dimethyl formamide (DMF) and tetrahydrofuran (THF), to form a homogeneous suspension that allows a better spread of the nanofiller in the polymeric matrix [3]. The nanocomposites based on polymers and carbon-based nanofillers [4,5,6] demonstrated improved performance against host matrices due to the enhancement of their optical, thermal, and mechanical properties [7].

Poly(vinyl chloride) (PVC) is one of the most widely used polymers in the world due to its low cost, easy processing, and good mechanical, corrosion, and chemical properties [8]. One of the major drawbacks of PVC is poor heat stability, which means that the construction materials based on this polymer are not stable during a fire. Some reports mention the usage of GO as filler in the polymeric matrix of PVC, which displays better behavior during thermal decomposition [1,9]. PVC/GO composites are interesting since the lone pairs of electrons located at the chlorine atom induce dipole–dipole interactions between the hydrogen and chlorine atoms stiffening the macromolecule backbone of the PVC. Furthermore, the interaction between GO and the polymer is facilitated by the chlorine atoms obtaining an active site that permits the binding of functional groups, thereby improving the composites’ properties, such as stability at high temperatures [1]. In this context, Deshmukh et al. [1] prepared and investigated the thermomechanical characteristics of membranes based on PVC and GO. The PVC/GO nanocomposite films were obtained by a colloidal blending method using THF as solvent. These composites show a morphology of interconnected micropores that induce an increase in thermal stability due to the strong interaction between the polymer and the carbon-based nanofiller. In another study, Deshmukh and his team [8] utilized the same method to obtain PVC/GO composites in the form of membranes, where instead of THF, DMF was used. These samples were reported to be used as electrode materials in supercapacitor cells [8]. Other strategies used for the preparation of the PVC/GO composites are as follows: (i) the phase inversion method, reported by Zhao [10], where hybrid membranes with enhanced hydrophilicity and mechanical properties and huge potential applicability in ultrafiltration membranes of wastewater were observed; (ii) casting solutions of PVC and GO in THF over a cation exchange resin powder to provide functional groups [7]. Recently, other applications of PVC/GO composites were detailed, for example, as an actuator for artificial muscle [11] and as an acoustic sensor [12]. The most used experimental methods for the characterization of these composite materials are thermogravimetry (TGA), scanning electron microscopy (SEM), FTIR spectroscopy, and Raman scattering [10,11,13].

In this study, GO was obtained by a more efficient and safer sono-chemical oxidation of graphite followed by specific separation-washing steps [14,15,16]. The contribution of this GO in increasing the fire protection was proven for wood materials covered with a layer containing GO, among other components [17]. Moreover, by a high-temperature action of a laser irradiation GO supported on a polymeric substrate, not burning was obtained when this system was placed between two glass sheets [18].

Taking into account the above progress, in this work, a new method for the preparation of PVC spheres covered with the GO sheets is reported. In order to prove the transformation of PVC grains into microspheres and to gain a better understanding of the chemical mechanism that occurs during the generation of such morphological structures, as well as the adsorption processes of GO layers on the PVC sphere surfaces, correlated studies of FTIR spectroscopy, Raman scattering, and X-ray photoelectron spectroscopy (XPS) are reported. The adsorption implications of GO layers on the surface of PVC spheres, in terms of the photoluminescence properties of this macromolecular compound, are also presented in this work. The potential of these composites as flame-retardant materials is reported with preliminary microscale combustion calorimetry (MCC) analysis.

## 2. Materials and Methods

Chemical compounds used in this work, i.e., PVC, lauroyl peroxide (LPO), hexyl ethyl cellulose (HEC), ethanol, THF, and DMF, were purchased from Sigma-Aldrich (St. Louis, MO, USA) without other purification processes. An oxidative–exfoliation method in the presence of 74.7 mL H_2_SO_4_ 85 wt.% and 8.3 mL H_3_PO_4_ 85% was used for the preparation of the GO sheets [14,15]. Detailed information about this method can be found in our previous studies [14,15,16]. In brief, graphite powder was sonochemical exfoliated using H_2_SO_4_ as solvent medium, KMnO_4_ as the oxidant agent, and H_3_PO_4_ as the protective agent against excessive oxidation. After steps that involved washing (with H_2_O_2_, H_2_O, HCl, and ethanol), sonication, and centrifugation–decantation, solid GO material was obtained by drying the specifically harvested well-exfoliated GO suspension [14,15]. Starting with 5 g of graphite, about 15 g of dried GO as ~100 cm^2^ irregular paper-like membranes (i.e., made up of connected two-dimensional GO nanosheets [14]) were obtained. This was easily dispersed in various solvents.

**Figure 1:**
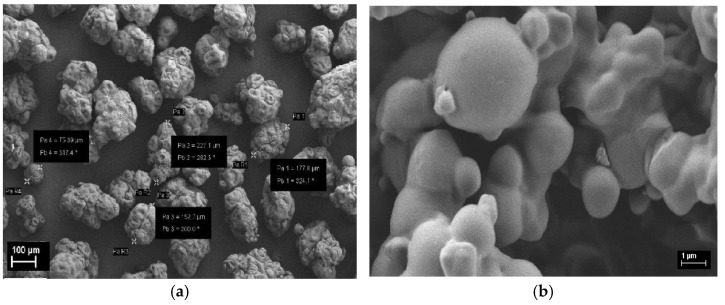
Scanning electron microscopy (SEM) micrographs of poly(vinyl chloride) (PVC) with the following morphological structures: grains with a size of 75–227 µm (**a**) and spheres with a diameter of 3.5 µm (**b**).

The PVC powder shows irregular grains that varied significantly in shape and size from 227 to 75 µm (Figure 1a). A method proposed by us to decrease the PVC grain size to 3.5 µm takes into account the principles of the suspension polymerization process. Early studies have shown that in the initiation stage of the VC polymerization reaction, radicals followed by macroradicals with various chain lengths, which form unstable microdomains, are generated [19]. These can subsequently be formed by nucleation primary particles with a size of ca. 0.1–1 µm. As polymerization takes place, the particles size grows. Depending on the conversion of polymerization reaction, grains with a size greater than 40 µm can occur [19]. In order to break the microdomains, a method proposed by us can be utilized, which consists of the interaction between PVC grains (0.4 g) dissolved under ultrasonication in THF (10 mL) and LPO (0.05 g in 10 mL DMF) in the presence of the aqueous solution HEC (0.1 g in 10 mL H_2_O) at 60 °C for 4 h. This reaction was stopped after 4 h by the rapid decrease in temperature at 25 °C in the presence of a large amount of H_2_O (500 mL), which resulted in white precipitation. This was filtered and dried until it was a constant weight, resulting in a white powder, which corresponds to the PVC sphere (Figure 1b). The generation of PVC spheres is a consequence of the formation of LPO radicals at 60 °C, which are responsible for the appearance of the PVC macroradicals. This process involves the breaking of microdomains and the generation of defects in the macromolecular chain as a result of the dehydrogenation reaction. 

In order to obtain PVC spheres coated with GO sheets, the above procedure was altered with the addition of 2, 4, 8, 12, 16, and 20 mg GO suspended in 10 mL DMF to the reaction mixture when the PVC/GO composites with a GO concentration of 0.5, 1, 2, 3, 4, and 5 wt.%, respectively, occurred. With the increase in GO concentration in the PVC/GO composite mass, the PVC spheres coated with the GO sheets showed a color range from light gray to dark gray. 

A Zeiss Gemini 500 scanning electron microscope (Oberkochen, Germany) was used in the SEM studies of PVC as grains and spheres, the GO sheets, and the PVC/GO composites.

Raman spectra of the PVC spheres and their composites with the GO sheets were recorded at the excitation wavelength of 514 nm with the T64000 model Raman spectrophotometer from Horiba Jobin Yvon (Pallaiseau, France) in backscattering geometry. 

IR spectra of the PVC spheres and their composites with the GO sheets were recorded with Bruker a Vertex 80 FTIR spectrophotometer (Billerica, US), in transmission geometry.

Photoluminescence (PL) spectra of the PVC spheres and their composites with the GO sheets were recorded with a Horiba Jobin Yvon Flurolog-3 FL3-22 spectrometer (Palaiseau, France) in right-angle geometry. 

XPS spectra of the PVC spheres and their composites with the GO sheets were recorded with a SPECS spectrometer (SPECS Gmbh, Berlin, Germany) which was endowed with an Al Kα source and a PHOBIOS 150 analyzer.

The MCC was developed by the Federal Aviation Administration (FAA) to screen research materials for a fireproof aircraft cabin. Standard flammability tests require kilograms of material; thus, the milligram (10^−6^ kg) samples of MCC are on the microscale by comparison. The MCC uses principles of analytical pyrolysis, combustion gas analysis, and flow calorimetry (i.e., pyrolysis–combustion flow calorimetry (PCFC)) to simulate the flaming combustion of plastics in a convenient laboratory test [20].

MCC analyses were performed with a microscale combustion calorimeter from Fire Testing Technology, UK, using the standard ASTM D7309-07, Method A [21], in order to determine the flammability characteristics of different kinds of materials using microscale combustion calorimetry. This method specifies that sample degradation takes place in a nitrogen atmosphere in a pyrolysis chamber, and, then, the fuel gases are introduced in a combustion chamber, where they are thermally oxidized to exhaustion [20].

Multiple MCC tests were conducted on the control sample in order to identify the optimal combination of the sample mass and the heat rate to ensure the lowering of oxygen concentration to 10 ± 3%, as recommended by the producers [20] and as the specialty literature states [22]. The sample mass was established at 14 mg, a heating rate of 2 K/s was set as the optimal rate, and two tests using these conditions were conducted for all of the samples. The samples were placed in an alumina cup measuring 6 mm in diameter and 4 mm in height and heated up to 750 °C in the pyrolysis chamber with a heating rate of 2 K/s, as mentioned above. In the combustion chamber, the temperature was kept constant at 900 °C to ensure that all volatiles were combusted.

## 3. Results and Discussion

### 3.1. Influence of GO Sheets on PVC Sphere Size

**Figure 2:**
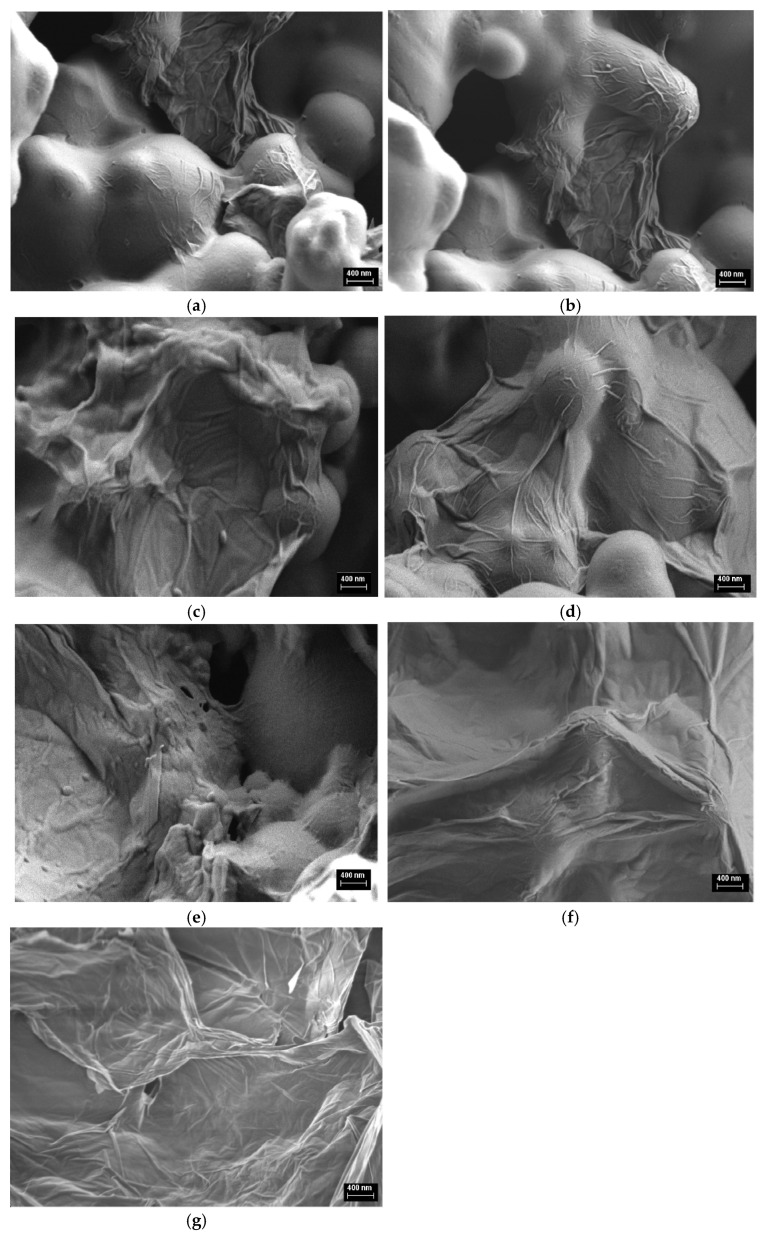
SEM micrographs of PVC/graphene oxide (GO) composites with a GO concentration equal to 0.5 wt.% (**a**), 1 wt.% (**b**), 2 wt.% (**c**), 3 wt.% (**d**), 4 wt.% (**e**), and 5 wt.% (**f**). GO sheets (**g**).

Figure 2 shows the SEM micrographs of the composites based on PVC spheres and GO sheets. According to Figure 2, the addition of various weights the GO sheets in DMF at the reaction mixture induces a decrease in the size of the PVC sphere between 3.5 and 0.7 µm, while the GO concentration in the PVC/GO composite mass increases from 0.5 to 5 wt.%.

An explanation for the decrease in the size of PVC grains as the concentration of GO sheets increases in the composition of the PVC/GO composites must consider the processes of the breaking of micro-domains from the PVC grains. The presence of GO sheets in the synthesis mixture prevents the coalescence process of small spheres into larger PVC spheres. In fact, this process is induced by the adsorption of the GO sheets on the PVC sphere surfaces.

### 3.2. Vibrational Properties of PVC Spheres and Their Composites with GO Sheets

**Figure 3:**
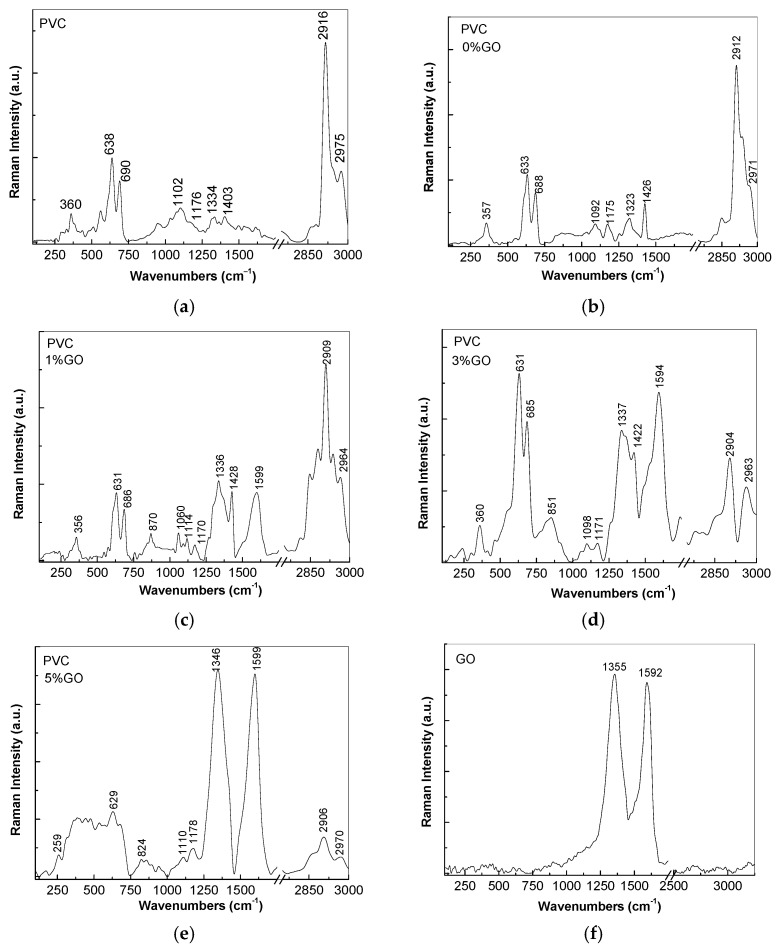
Raman spectra of the PVC grains (**a**), the PVC spheres (**b**), and their composites with GO when the concentration of GO sheets is equal to 1 wt.% (**c**), 3 wt.% (**d**), and 5 wt.% (**e**). GO sheets (**f**).

In order to confirm that morphological structures (as shown in Figure 1 and Figure 2) corresponding to the PVC spheres and their composites with GO, the vibrational properties of these are shown by Raman scattering and FTIR spectroscopy. Thus, Figure 3a shows the Raman spectrum of the commercial PVC powder in the form of grains. The main Raman lines of the PVC grains as well as their assignment are shown in Table 1 [22,23,24,25].

In contrast with the Raman spectrum of PVC grains, in the case of the Raman spectrum of PVC spheres, the following changes are reported: (i) a down-shift of the Raman lines from 360, 638, 1102, 1334, 2916, and 2976 cm^−1^ to 357, 633, 1092, 1323, 2912, and 2971 cm^−1^, respectively; (ii) an up-shift of the Raman line from 1403 cm^−1^ to 1426 cm^−1^. Recently, such shifts in the position of Raman lines of the macromolecular compound were reported in the case of membrane-type PVC/GO composites, obtained by the method of mixing solutions of PVC in THF and GO in DMF [26]. A characteristic of the Raman spectra of PVC as grains and spheres is the ratio between the intensities of the Raman lines situated in the spectral ranges of 600–750 and 2850–3000 cm^−1^, where the maxima of most of the intense Raman lines can be found at 638 and 2916 cm^−1^ (Figure 3a) and 633 and 2912 cm^−1^ (Figure 3b), equal to 0.41 and 0.39, respectively. The decrease in the intensity of the Raman line situated in the spectral range of 2900–2930 cm^−1^ was reported in the case of the membranes based on PVC/GO composites as a result of the appearance of defects, in the PVC repeating units, of the type ClCH=CH– and/or –CH=CCl– [26]. The appearance of these defects in the PVC macromolecular chain was also reported in the case of other composites, such as PVC/carbon nanotubes [27] and PVC/graphene [28]. In all cases, the above behavior of the Raman line situated in the spectral range of 2900–2930 cm^−1^ was attributed to catalytic activity of carbon nanoparticles in the PVC partial dehydrogenation process. Figure 3f shows the Raman spectrum of the GO sheets, which is characterized by two bands with a maxima at 1592 and 1355 cm^−1^, labeled as the G and D bands, respectively, with them being assigned to the vibrational modes of the radial hexagonal carbon rings and E_2g_ at the Brillouin area center [29]. The intensity ratio of the D and G Raman lines of the GO sheets represents a measure of disorder; in our case, this is ≅ 1. According to Figure 3b–d, as the concentration of GO sheets in the PVC/GO composite mass increases, the main changes in the Raman spectra of these materials consist of the following: (i) the change in the ratio in terms of the relative intensities of the Raman lines situated in the 600–750 and 2850–3000 cm^−1^ spectral ranges, with a maxima of the most intense Raman lines peaking at 633 and 2912 cm^−1^ (Figure 3b), 631 and 2909 cm^−1^ (Figure 3c), and 631 and 2904 cm^−1^ (Figure 3d) from 0.39 (Figure 3b) to 1.63 (Figure 3d) and from 0.39 to 1.63; (ii) the gradual increase in the intensity of the D and G Raman bands of the GO sheets simultaneously with a down-shift of the D band from 1355 to 1346 cm^−1^ and an up-shift of the G band from 1592 to 1599 cm^−1^.

**Figure 4:**
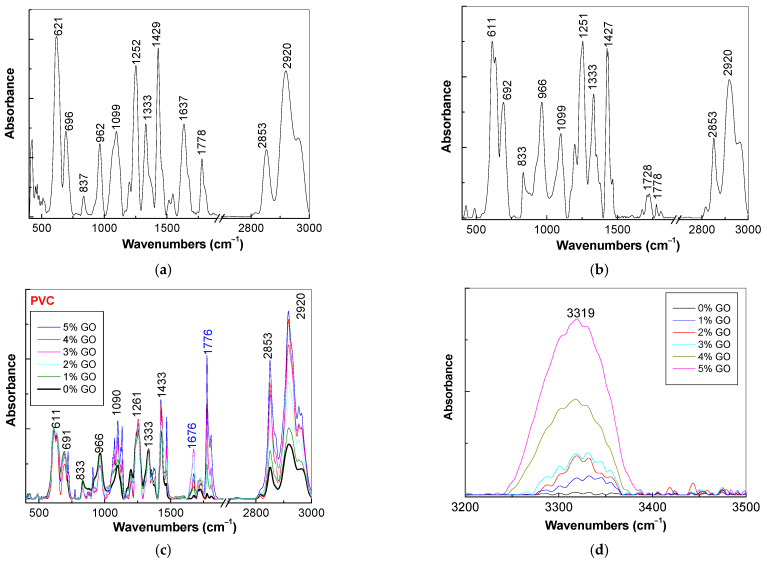
IR spectra of the PVC as grains (**a**) and spheres (**b**) as well as (**c**) their composites with PVC spheres (black curve), with a concentration of GO sheets equal to 1 wt.% (green curve), 2 wt.% (blue curve), 3 wt.% (magenta curve), 4 wt.% (red curve), and 5 wt.% (dark blue curve). Figure (**d**) shows the IR spectra of the GO sheets and their composites with PVC in the 3200–3500 cm^−1^ spectral range.

Figure 4 shows the IR spectra of PVC as grains and sphere as well as their composites based on the PVC spheres coated with GO sheets. The assignment of the main IR bands of the PVC grains in Figure 4a are shown in Table 2 [30,31,32,33]. 

The following changes are noted in the IR spectrum of the PVC spheres: (i) a down-shift of the IR bands from 621, 696, and 837 cm^−1^ (Figure 4a) to 611, 692, and 833 cm^−1^, respectively (Figure 4b); (ii) an up-shift of the IR bands from 962 and 1252 cm^−1^ (Figure 4a) to 966 and 1256 cm^−1^, respectively (Figure 4b); (iii) the appearance of an IR band with a maximum of 1728 cm^−1^ as a result of the generation of new C=O bonds during the preparation of the PVC spheres; and (iv) an increase in the absorbance of the IR band at 1333 cm^−1^ simultaneously with the disappearance of the IR band with the maximum of 1637 cm^−1^. In this context, we noted that shifts of the IR bands of PVC were often reported in the case of composites based on PVC and carbon nanoparticles, such as multi-walled carbon nanotube [34] and graphene [28]. 

**Scheme 1:**
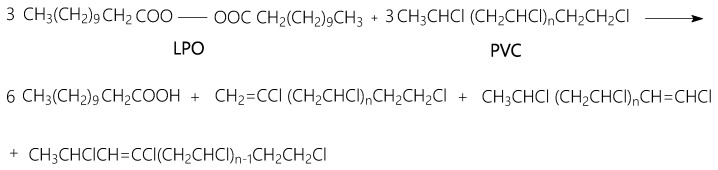
Interaction of PVC grains with lauroyl peroxide (LPO).

These changes can be explained when taking into account Scheme 1. In our opinion, Scheme 1 explains (i) the presence of new C=O bonds as a consequence of the generation of the compound CH_3_(CH_2_)_9_CH_2_COOH; (ii) the increase in the intensity of the IR band at 1333 cm^−1^ assigned to the vibrational mode of the bending of CH_2_ groups as a consequence of a partial transformation of the CH_3_–CHCl– bonds in the CH_2_=CCl– bonds; (iii) the shift in the IR bands localized in the 600–900 cm^−1^ range assigned to the vibrational modes of the wagging of C-H bonds of PVC with a syndiotactic structure, the stretching of C–Cl bonds of PVC with an isotactic structure, and the stretching of C–Cl bonds as a result of the generation of defects on the PVC backbone chains of the CH_2_=CCl–, –CH=CHCl, and –ClCH=CCl– bonds.

**Scheme 2:**
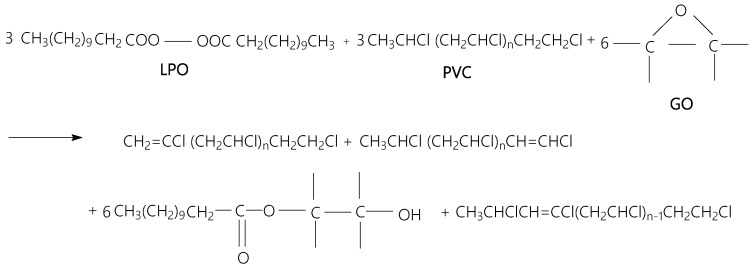
Interaction of PVC grains with LPO and GO sheets.

These changes must be understood in terms of the appearance of some of the ClCH=CH–, CH_2_=CCl–, and/or –CH=CCl– units as a result of the partial dehydrogenation of the PVC macromolecular chains, as shown in Scheme 1. As shown in our previous study, the main IR bands of the GO sheets are situated at 1032, 1163, 1212, 1414, 1599, 1724, and 3594 cm^−1^, and they are assigned to the vibrational mode of the stretching of C=C bonds, C–O in the ether group, OH groups, the deformation of OH and COOH groups, H_2_O molecules on the GO sheet surfaces, the stretching of C=O in COOH and CHO groups, and the stretching OH in the free H_2_O molecules, respectively [16,28]. According to Figure 4c,d, as the GO sheet concentration in the PVC/GO composite mass increases, the following can be observed: (i) an increase in the absorbance of IR bands peaking at 1776 and 3319 cm^−1^; (ii) a change in the ratio of the absorbances of the bands localized in the spectral ranges of 600–750 and 2750–3250 cm^−1^ with the maxima of IR bands of high absorbance at 611 and 2920 cm^−1^, from 1.28 (black curve in Figure 4c) to 0.36 (blue curve in Figure 4c) when the GO concentration is equal to 0 and 5 wt.%, respectively. These changes can be explained when Scheme 2 is considered.

**Scheme 3:**
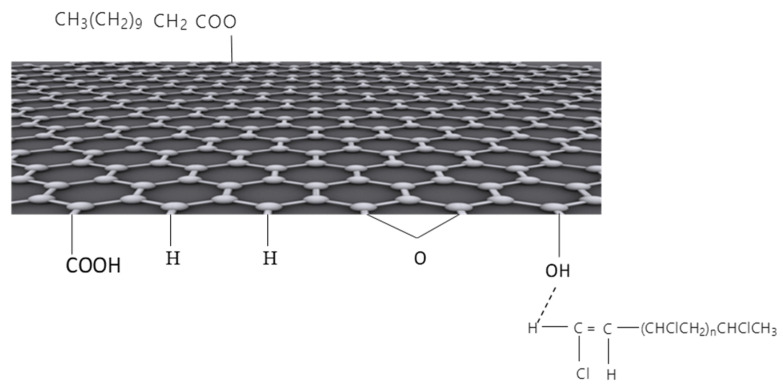
Adsorption of GO sheets on PVC spheres.

Scheme 2 explains the increase in the absorbance of the IR bands that peaked at 1776 and 3319 cm^−1^ as a consequence of the appearance of additional defects of the C=O bonds in the ester groups and OH bonds, respectively, [35,36] on the GO sheet surfaces. The adsorption of the GO sheets on PVC spheres involves the establishment of new hydrogen bonds between the hydroxyl group of the GO sheets and the macromolecular chains of PVC, as shown in Scheme 3, a process which prevents the aggregation of PVC spheres.

### 3.3. XPS Spectra of PVC Spheres and Their Composites with GO Sheets

**Figure 5:**
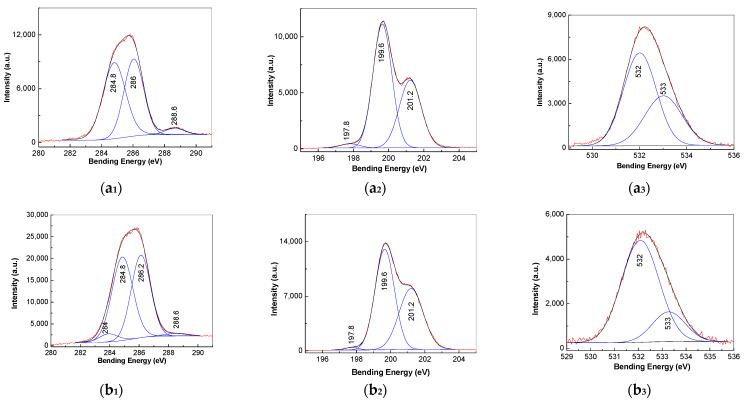

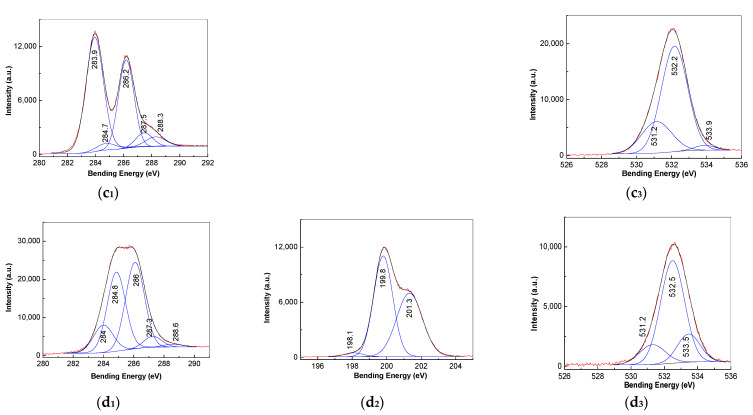
X-ray photoelectron spectroscopy (XPS) C1s spectra of PVC grains (**a_1_**), PVC spheres (**b_1_**), GO sheets (**c_1_**), and PVC spheres coated with GO sheets (**d_1_**). XPS Cl2p spectra of PVC grains (**a_2_**), PVC spheres, (**b_2_**) and PVC spheres coated with GO sheets (**d_2_**). XPS O1s spectrum of PVC grains (**a_3_**), PVC spheres (**b_3_**), GO sheets (**c_3_**), and PVC spheres coated with GO sheets (**d_3_**).

Additional information that confirms Scheme 2 and Scheme 3 is shown by XPS spectroscopy in Figure 5. Figure 5a_1_ highlights three peaks in the case of the XPS C1s spectrum of the PVC grains at 284.8, 286, and 288.6 eV, which are assigned to the C–C and C–H bonds in the entities of –CH=CH– type and the C–Cl bonds in the entities of –(CHCl–CH_2_)– and –COO– bonds, respectively [33,37]. Figure 5a_2_ shows two peaks of high intensity in XPS Cl2p with a maxima of 201.2 and 199.6 eV, labeled Cl 2p^1/2^ and Cl 2p^3/2^, respectively, which are attributed to the C–Cl bonds [38]. The peak of low intensity with a maximum of 197.8 eV is assigned to the hydrogen bonds [39]. The deconvolution of the XPS O1s spectrum of PVC grains (Figure 5a_3_) highlights two peaks at 532 and 533 eV, which are assigned to the C–C=O and C–O–C bonds, respectively [38]. In contrast with the XPS C1s spectrum of the PVC grains (Figure 5a_1_), in the case of Figure 5b_1_, the appearance of a new peak at 284 eV assigned to the sp^2^ C atoms can be observed, a fact that confirms the appearance of the double bonds on the PVC backbone chain highlighted in Scheme 1 as CH_2_=CCl–, –CH=CCl–, and –CH=CHCl.

An increase in the ratio of intensity of the peaks at 532 and 533 eV from 1.82 (Figure 5a_3_) to 2.95 (Figure 5b_3_) is reported when the transformation of the PVC grains into the PVC spheres takes place. This can be a consequence of the adsorption of CH_3_(CH_2_)_9_CH_2_COOH on the PVC sphere surfaces. Five peaks are noted in the XPS C1s spectrum of the GO sheets (Figure 5c_1_) at 283.9, 284.7, 286.2, 287.5, and 288.3, eV, which were assigned to the sp^2^ C atoms in the C=C bond, the sp^3^ C atoms in the C–C and C–H bonds, the C–O in hydroxide groups, C–O in the epoxide groups, and the O–C=O bond in the carboxylic groups, respectively [40]. According to Figure 5c_3_, the XPS O1s spectrum of the GO sheets was deconvoluted into three peaks at 531.2, 532.2, and 533.9 eV, assigned to the C=O bond in carbonyl or ester, the C–O–C bond in the epoxy and hydroxyl groups, and free water molecules, respectively [41]. Figure 5d_1_ shows the XPS C1s spectrum of the PVC spheres coated with the GO sheets, where both the peaks belonging to the PVC sphere, such as those at 284, 284.8, 286, and 288.6 eV, and the peak of the GO sheets at 287.3 eV are observed. A careful analysis of Figure 5b_1_,d_1_ highlights a variation in the position of the peak from 286.2 eV (Figure 5b_1_) to 286 eV (Figure 5d_1_) accompanied by a change in the ratio of intensity of the peaks at 284.6 and 286 eV form 0.98 (Figure 5b_1_) to 0.85 (Figure 5d_1_). This result is a consequence of the presence of the C–O bonds in the hydroxide groups that are generated onto the GO sheet surfaces, according to Scheme 2 and Scheme 3. The careful analysis of Figure 5b_3_,d_3_ indicates that the ratio of intensities of the peaks at 532 and 531 eV decreases from 0.31 to 0.2, a fact that indicates an increase in the C–O–C bonds from the ester groups on the GO sheet surfaces, generated as shown in Scheme 2.

### 3.4. Photoluminescence of PVC Spheres and Their Composites with GO Sheets

**Figure 6:**
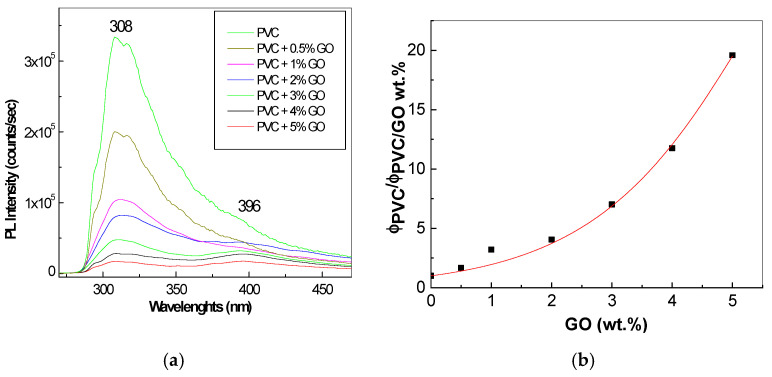
Photoluminescence (PL) spectra of PVC and composites with GO when the GO sheet concentration is equal to 0.5 wt.%, 1 wt.%, 2 wt.%, 3 wt.%, 4 wt.%, and 5 wt.% (**a**). Dependence of the ratio between PL band intensity of PVC spheres and PVC/GO composite with the concentration of GO sheets (**b**).

The influence of the GO sheet adsorption on PL of the PVC spheres is shown in Figure 6.

Using the excitation wavelength of 240 nm, a PL band with a maximum of 325 nm is reported in the case of the PVC spheres. A PVC PL quenching process is observed to be induced by the increase in the GO sheet concentration in the PVC/GO composite weight. The strength of the PL quenching effect due to the presence of the GO sheets can be highlighted in terms of its dependence on the ratio between PL band intensity of the PVC spheres (ϕ_F_^PVC^) and the PVC/GO composite (ϕ_F_^PVC-GO^) with the concentration of the GO sheets (labeled in the equation below as [GO]). This curve fits with the following equation:

ϕ_F_^PVC^/ϕ_F_^PVC-GO^ = (1 + k_q_ τ_F_^0^ [GO]) × exp (Nν[GO])

where N, ν, k_q_, and τ_F_^0^ correspond to the Avogadro’s number, the static quenching volume, the bimolecular quenching rate constant, and the fluorescence lifetime in the absence of a quencher, respectively. The values for k_q,,_ τ_F_^0^, and Nν in the above equation are equal to 0.9871, 0.5764, and 0.3244, respectively. The above equation describes an exponential growth of the static quenching effect due to the formation of ground-state complexes and excited-state exciplexes. 

The change in the ratio of the relative intensity of the PL bands with a maxima of 308 and 396 nm from 4.44 to 1 when the concentration of the GO sheets is equal to 0 and 5 wt.%, respectively, indicates the formation of a new luminescent center, which can be defects induced both during the generation of the PVC spheres and the adsorption of GO sheets on PVC particle surfaces.

### 3.5. Potential of PVC Spheres Coated with GO Sheets as Flame-Retardant Materials

**Figure 7:**
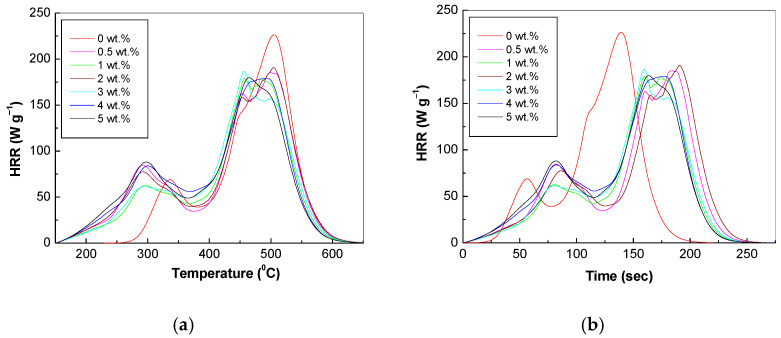
Dependence of heat release rate (HRR) on temperature (**a**) and time (**b**) for samples labeled as PVC spheres (red curve) and composites based on the PVC spheres and GO sheets with the GO concentration equal to 0.5 wt.% (magenta curve), 1 wt.% (green curve), 2 wt.% (wine curve), 3 wt.% (cyan curve), 4 wt.% (blue curve), and 5 wt.% (black curve).

Figure 7 shows results of the MCC analysis carried out using the PVC spheres and their composites with GO sheets. The thermal decomposition of the PVC grains was reported to takes place in two stages [42,43,44,45,46]. The first stage takes place in the temperature range of 260–360 °C, where HCl is mainly formed after decomposition, as well as benzene and, in very small quantities, aromatic hydrocarbons and aromatic alkyl, respectively [44]. The second stage takes place in the temperature range of 420–520 °C, where a higher weight of aromatic hydrocarbons and aromatic alkyl simultaneous with very small amounts of HCl and benzene occur [44]. In the case of PVC spheres, Figure 7a highlights the two peaks of the heat release rate (HRR) at 335.88 °C and 505.64 °C, the latter being accompanied by a shoulder at 455.85 °C. The values of the HRR at the temperatures of 335.88, 455.85, and 505.64 °C are equal to 68.92, 145.04, and 226.32 W g^−1^, respectively. Figure 7b highlights the increase in the time at which the peak of heat release rate (PHRR) is reached as a result of the increase in GO sheet concentration on the PVC spheres. According to Table 1, the heat release capacity (HRC) of the PVC spheres is 168.13 J g^−1^ K^−1^, a value that is smaller in comparison with that reported for the PVC grains—175 J g^−1^ K^−1^ [20]. 

In MCC analysis, a microlevel analysis regarding fire behavior of construction materials, the HRC is the most important parameter. Low levels of the HRC indicate a low level of inflammability in the MCC test and a low level of fire risk in real life [47]. Considering that the char yield percentage represents the unburned quantity of combustibles from a material treated with substances that reduce inflammability, it is expected that the material with the highest char yield percentage will have a smaller heat release rate when burning [48], thereby resulting in a correspondence relation between HRC and char yield. Following the numerical analysis, there are observable improvements from the point of view of char yield and from the point of view of HRC for the composite samples containing 2–5 wt.% GO. The lowest HRC value is calculated for the 5 wt.% PVC-GO sample consisting of 143.17 J/(g*K), which is 14.85% lower than the HRC of the control sample of 168.13 J/(g*K). This sample also has the highest char yield, 8.34%, which is 2.1% higher than that of the control sample. There are also observable improvements from a temporal point of view, where there is both an approximate 26.5-s delay of the first peak for all composite samples when compared to that of the control PVC sample and a delay in the appearance of the PHRR by 20 to 51.5 s, as shown in Figure 7b. Data from the literature show that the THR is independent of the char yield value for halogenated polymeric compounds [49], and this fact is also visible in Table 3. The MCC parameters were correlated to the conventional small- and medium-scale fire performance tests. It was shown that for pure polymers, the HRC is well correlated with the heat release rate for the peak heat (PHRR, kW/m^2^) measured by cone calorimetry at a high heat flux where the pyrolysis is relatively complete [50,51]. The limit oxygen index (LOI) decreases as the HRC increases [50]. There was an established range of HRC values to support the evaluation of the UL94 classification of pure polymers [51]. As a result, the MCC remains an appropriate small-scale apparatus for rapid flammability screening of flame-retardant (FR) compounds [49].

Summarizing the data shown in Table 3 and Figure 7, we can conclude that these preliminary results of MCC sufficiently show the following: (i) an increase is observed in the time at which the maximum heat release rate with the GO sheet concentration is reached in the PVC/GO composite mass; (ii) compared to the HRC value of PVC spheres, the lowest value in the case of composites based on PVC spheres covered with GO sheets is reported for the samples that have a GO concentration equal to 5 wt.%.

## 4. Conclusions

In this work, a new method of transforming PVC grains into PVC spheres, as well as the preparation of composites based on PVC spheres and GO sheets, was reported. The results reported by Raman scattering, FTIR spectroscopy, XPS spectroscopy, photoluminescence, and MCC analysis allow us to draw the following conclusions: (i) Interaction between the PVC grains with a size of 75–227 μm and HEC and LPO at a temperature of 60 °C results in PVC spheres with a size of ~3.5 μm; this reaction involves the appearance of new defects on the PVC backbone chain of the bonds ClCH=CH–, CH_2_=CCl–, and/or -CH=CCl– as a result of the partial dehydrogenation of the PVC. (ii) The addition of the GO sheets in the reaction mixture of PVC spheres leads to composites based on PVC spheres with a size <3.5 to 0.7 μm coated with GO sheets; this result can be explained when taking into account that the presence of GO sheets in the synthesis mixture prevents the coalescence process of small spheres into PVC larger spheres. The resulting composites show GO sheets with a higher weight in the ester and in hydroxyl groups in comparison with the initial state of the GO sheets. Adsorption of the GO sheets on the PVC spheres was observed to occur via hydrogen bonds. (iii) The presence of GO sheets on the PVC sphere surface induces a PVC PL quenching process. (iv) The coating of the PVC spheres with GO sheets induces an increase in the time at which the maximum heat release rate is reached; the composites based on PVC spheres covered with GO sheets, which have a GO concentration equal to 5wt.%, show a lower HRC value. These preliminary results highlight that composites based on PVC spheres covered with GO sheets can be promising flame-retardant agents. In order to improve these results, further research regarding the functionalization of GO sheets with nitrogen- and phosphor-based compounds and their deposition on the PVC spheres is in progress in order to optimize the performances of these composites materials as flame-retardant agents.

## Figures and Tables

**Table 1:** Vibrational modes observed by Raman spectroscopy in poly(vinyl chloride) (PVC) grains.

ν (cm^−1^)	Assignments [22,23,24,25]
360	C–Cl in the trans configuration of polymer
638–690	C–Cl stretching
1102–1179	C–C stretching
1334–1403	C–H symmetrical stretching in CH_2_ group
2916	C–H asymmetrical stretching in CH_2_ group
2975	C–H asymmetrical stretching in CH_3_ group

**Table 2:** Vibrational modes observed by FTIR spectroscopy in PVC grains.

ν (cm^−1^)	Assignments [30,31,32,33]
621	wagging of C–H bonds of syndiotactic structure of PVC
696	stretching C–Cl bond of isotactic structure of PVC
837	rocking of C–H bond in CH_2_ group
962	wagging of C–H bond in polymer with trans configuration
1099	deformation of C-H bonds
1252	rocking of C–H bond in polymer with trans configuration

**Table 3:** Main parameters of microscale combustion calorimetry (MCC) analysis: char yield—percentage quantity of remanent residue, decomposition rate—percentage quantity of sample that was used in the combustion process, HRC—heat release capacity, THR—total heat release; PHRR—peak of heat release rate, T_PHRR_—temperature of maximum pyrolysis speed, time—the moment at which PHRR is reached.

Sample	Mass (mg)	Residues (mg)	Char Yield (wt.%)	Decomposition Rate (%)	HRC (J g^−1^K^−1^)	THR (kJ g^−1^)	PHRR (W g^−1^)	T_PHRR_ (°C)	Time (s)
PVC-GO 0 wt.%	14.02	0.88	6.25	93.75	168.13	14.27	226.32	505.64	139.50
PVC-GO 0.5 wt.%	14.04	0.92	6.55	93.45	190.61	15.83	185.12	500.27	184.00
PVC-GO 1 wt.%	14.03	1.13	8.05	91.95	173.76	14.87	178.97	457.11	159.50
PVC-GO 2 wt.%	14.00	0.91	6.50	93.50	159.88	16.01	190.74	504.91	191.00
PVC-GO 3 wt.%	14.07	1.01	7.18	92.82	148.78	15.18	186.71	456.24	159.50
PVC-GO 4 wt.%	14.10	1.05	7.42	92.58	145.29	16.09	179.05	494.26	178.00
PVC-GO 5 wt.%	14.08	1.17	8.34	91.66	143.17	15.87	179.84	464.84	163.50

## Data Availability

The data presented in this study are available on request from the corresponding author.
